# Influence of national culture on mentoring relationship: a qualitative study of Japanese physician-scientists

**DOI:** 10.1186/s12909-021-02744-2

**Published:** 2021-05-25

**Authors:** Haruo Obara, Takuya Saiki, Rintaro Imafuku, Kazuhiko Fujisaki, Yasuyuki Suzuki

**Affiliations:** 1 Medical Education Development Center, Gifu University, 1-1 Yanagido, Gifu, 501-1194 Japan; 2 Department of Medicine, Okinawa Prefectural Chubu Hospital, 281 Miyazato, Uruma, 904-2243 Japan

**Keywords:** Mentoring, Physician-scientist, Japan, Cultural dimension, Hofstede, Globalization, Qualitative study

## Abstract

**Background:**

Nurturing of physician-scientists is an important mission of academic medical institutes. Although the importance of mentorship in developing future physician-scientists internationally is well established, not much information is available about how they are mentored and how national cultures influence the mentoring relationship. This study explores the cultural characteristics of mentoring relationships between senior mentors and junior mentees of Japanese physician-scientists.

**Method:**

A qualitative approach has been employed to explore mentoring relationships of Japanese physician-scientists from cultural viewpoints, through semi-structured interviews with 17 mentees who had the experience of working overseas as post-doctoral research fellow. The reflection of their experiences and the perception of mentoring relationships before going abroad were thematically analyzed by applying Hofstede’s model of six cultural dimensions as a theoretical framework.

**Results:**

Twelve characteristic themes for mentoring were observed, including trustworthy dependency on mentor, embracing paternalistic mentoring, mentee’s initiative within expectations of mentor based on power distance, a sense of loyalty to mentor/organization/colleagues, family-like relationship with mentor based on collectivism, sense of security on being led by mentor through uncertainty avoidance, motivation by role modeling for the competitive academic world, and adaption of female mentee/mentor to a male-dominated academic structure based on masculinity, a long-term relationship between mentor and mentee, receiving advice for organizational continuity based on long-term orientation, putting work before leisure, and friendly relationship between mentor and mentee outside of work based on indulgence.

**Conclusions:**

This study identified the characteristic mentoring relationships of postgraduate mentees of Japanese physician-scientists. Considering the importance of mentoring for physician-scientists in a globalized society, understanding the characteristics of national cultures would help in ensuring culture-sensitive mentoring and would contribute to the development of academic medicine.

## Background

Nurturing physician-scientists who greatly contribute to the development of medicine is indispensable for academic medical institutions [1]. However, decreased retention in academia of early career physicians has become a global challenge [2, 3] so much so that the term “endangered” is now used for them [4]. Japan, a research-producing nation in medicine, is facing a critical situation. The number of research articles published has been slow as compared to other major research focused nations in recent years [5–7], and the number of young physician-scientists has been decreasing [8].

Mentoring to nurture physician-scientists has garnered attention as an educational strategy to address this critical situation [9]. Mentoring plays an important role in career development [10], career satisfaction [11], performance enhancement, and identity formation [12], of the physician-scientist. Classically, mentoring has been defined as a process through which an experienced, highly regarded, and empathetic person (the mentor), guides another individual (the mentee) in the development and re-examination of their ideas, learning, and personal and professional development. The mentor, who often works in the same organization or field as the mentee, achieves this by privately providing career functions (such as sponsorship, exposure and visibility, coaching, challenging assignments, and protection to the protégé), and psychosocial functions (such as role modeling, acceptance and confirmation, counseling, and friendship) for mentees [13–15]. The more contemporary view of mentoring is that of a mentoring team, which includes people who are senior, junior, and peers, both within and outside the organization or field. Recently, mentoring in medicine has been found to be a process that is entwined, individualized, evolving, context-specific, goal-sensitive, and relational, as well as organization-, mentee-, and mentor-dependent [14]. A qualitative study conducted in North America found that successful mentoring relationships are characterized by reciprocity, mutual respect, clear expectations, personal connection, shared values, and effective actions that include, providing career guidance, offering emotional support, and focusing on work-life balance [16].

Most previous research on mentoring in academic medicine has been conducted in Western countries, and focused on intrapersonal and interpersonal factors affecting mentoring relationships [17]. In addition to these factors, Sambunjak proposed that environmental factors, such as culture, economy, and politics, also influence mentoring relationships [17]. Shamim elaborated on the issues associated with mentoring in South Asia, including cultural differences, gender issues, languages, socio-political views, and religion [18]. By exploring cultural influences in the development and maintenance of mentoring relationships in Malawi, Sawatsky et al. indicated that cultural values and norms are important aspects of mentoring in academic medicine [19]. A cross-sectional survey of Japanese physician-scientist mentees in academic medical centers, elaborated on mentor-led mentorship and stated that mentees expect assistance in long-term career planning [20]. Understanding the characteristics of different cultural backgrounds and values is not only beneficial to ensure mutual understanding between mentors and mentees but also leads to sharing more diverse ways of mentoring [21].

While it is established that cultural diversity would have an impact on mentoring, our understanding is that no specific frameworks of culture have been applied to analyze the cultural differences in mentoring in academic medicine so far. Previous scholarly works have offered many definitions of culture [22]. Hofstede defines culture as “the collective programming of the mind that distinguishes the members of one group or category of people from another.” [23] Through empirical studies conducted across 70 different countries, Hofstede identified and validated six dimensions of culture: 1) power distance, 2) individualism versus collectivism, 3) uncertainty avoidance, 4) masculinity versus femininity, 5) long-term orientation, and 6) indulgence versus restraint [23]. Hofstede’s model of six cultural dimensions has been widely used as a cultural framework for social sciences and cross-cultural studies [23]. These dimensions have also been used in cross-cultural studies in healthcare and medical education, such as, qualitative studies of physicians attitude toward antimicrobial prescribing [24], physician communication [25], and difficulties faced by tutors in problem-based learning [26]. Among these dimensions, power distance and individuality have been emphasized as relevant dimensions for mentoring relationships [27]. However, Hofstede’s dimensions have not been used as the theoretical framework for research to analyze mentoring relationships in academic medicine [27]. Exploring mentor-mentee relationships from the perspective of national culture could help to broaden the understanding of mentoring relationships and evidence for the need to consider cultural context in further research in mentoring, which in turn may contribute to the reformation of the training and retention of physician-scientists.

This study explores how cultural characteristics influence the relationship between senior mentors and junior mentees, in the context of career development of Japanese physician-scientists belonging to academic institutions.

## Methods

### Context

Most physician-scientists in Japan are a part of clinical research organizations or departments. These consist of a group of researchers or clinicians*,* including PhD trainees for several years after graduating from medical school and the residency training, and are often known as *Ikyoku* [7, 28, 29]. Studying abroad as a postdoctoral fellow is a typical progression in the career path and is considered important for career development as physician-scientists after the initial research training in Japan [30]. Confucianism in Japan requires respect and obedience toward elderly people who have historically responded with highly paternalistic attitudes toward younger people. Japanese organizations tend to be extremely hierarchical and rigidly organized [31, 32]. Senior, experienced mentors from the mentee’s *Ikyoku* offer a variety of mentoring, including mediation, negotiation, and support. The mentoring relationship usually continues not only during their training period in Japan, but also during and after studying abroad while they belong to the same *Ikyoku*. In terms of gender, female physician-scientists pursuing academic careers are still a minority in Japan, although the numbers are gradually increasing [33]. Among OECD countries, Japan has the lowest number of female doctors, about 21% [34].

### Study design

A qualitative thematic investigation [35] was employed to explore the cultural characteristics of the mentoring relationships of Japanese physician-scientists, by interviewing the mentees who pursued post-doctoral research fellowship abroad. The research is based on their reflection and perception of mentoring experiences in Japan.

## Theoretical framework

Hofstede’s cultural dimensions [23] were used as the framework to analyze and interpret mentoring relationships. The definitions, characteristics, and scores are described in Table 1 [23, 36].

**Table 1:** Six cultural dimensions as suggested by Hofstede with a few modifications [23, 36]

Dimensions (scores)	Definitions	Countries’ scores (0–100)				
		Japan	China	South Korea	UK	USA
Power distance, large vs small	The degree to which the less powerful members of a society accept and expect that power is distributed unequivocally. In cultures with large power distance, junior members are expected to wait for instructions from their supervisors, whereas in a society with small power distance, supervisors expect their juniors to take the initiative themselves.	54	80	60	35	40
Individualism (high) vs Collectivism (low)	The degree to which people in society are integrated into groups. Individualism is defined as a preference for a loosely-knit social framework in which individuals are expected to take care of themselves and their immediate families, whereas collectivism represents a preference for a tightly-knit social framework in which individuals expect their relatives or members of a particular in-group to look after them in exchange of unquestioning loyalty.	46	20	18	89	91
Uncertainty avoidance, high vs low	The degree to which the members of a society feel uncomfortable with uncertainty and ambiguity.	92	30	85	35	46
Masculinity (high) vs femininity (low)	Masculinity stands for a preference in society for achievement, heroism, assertiveness, and material rewards for success. Femininity stands for a preference for cooperation, modesty, caring for the weak, and quality of life.	95	66	39	66	62
Long term orientation (high) vs short term orientation (low)	The extent to which the fostering of virtues is oriented toward future rewards, in particular, perseverance and thrift. Long term orientation is a tendency to look to the future and make decisions about what to do now. Short-term orientation is to focus on the present because the future is unpredictable.	88	87	100	51	26
Indulgence (high) vs restraint (low)	Indulgence implies a society that allows relatively free gratification of basic and natural human initiatives related to enjoying life and having fun. Restraint implies a society that suppresses gratification of needs and regulates it by means of strict social norms.	42	24	29	69	68

### Participants

Using convenience sampling, 17 Japanese physician-scientists who were pursuing postdoctoral research fellowship in foreign countries (Table 2) were recruited for the study. They represented typical physician-scientists who aspire to continue working in the field of academic medicine. Ten participants were interviewed while they were studying in the United States as research fellows before they returned to Japan. Seven participants were interviewed in Japan after they completed research fellowships abroad (Table 2). Three of the participants were female physicians and 14, male. The mean age of the participants was 39.2 years and the mean years of physician experience was 14.2 years. Fifteen participants studied in USA and two in France. All participants had both MD and PhD degrees.

**Table 2:** Demographics of participants

Code	Gender	Age	PGY	Specialty
1	F	30s	10	Psychiatry
2	M	30s	10	Anesthesiology
3	M	30s	8	Internal Medicine
4	F	30s	12	Internal Medicine
5	M	30s	7	Anesthesiology
6	M	30s	10	Internal Medicine
7	M	30s	12	Internal Medicine
8	M	30s	9	Internal Medicine
9	M	30s	8	Dermatology
10	M	30s	7	Ophthalmology
11	M	40s	21	Pediatrics
12	M	30s	29	Internal Medicine
13	F	50s	26	Internal Medicine
14	M	50s	13	Pediatrics
15	M	40s	21	Otorhinolaryngology
16	M	40s	23	Internal Medicine
17	M	40s	15	Psychiatry

All participants were Japanese

### Data collection

Participants completed a questionnaire that collected their demographic information before the interview. Semi-structured interviews were individually conducted in Japanese for about 60 min by the first author (HO), in a private room where privacy was ensured. Moreover, the researcher tried to create an open and friendly atmosphere so that the participants would feel safe and comfortable in the interview situation. Based on the interview guide (Table 3), we explored participants’ careers as mentees and their experiences and perceptions of a significant mentoring relationship with a Japanese mentor in Japan before they studied abroad. Interview data were digitally recorded, transcribed, and analyzed.

**Table 3:** Interview guide

Tell me about a senior mentor who had an influence on your career development as an academic physician-scientist till now.	
1. How was the relationship with this mentor?	
2. What and how did the mentor actually act or give advice to you?	
3. How did you feel about the mentor’s actions or advice?	

### Data analysis

The research team comprised five researchers from different backgrounds: an internist (HO), a general practitioner (TS), an education researcher (RI), a medical educator (KF), and a pediatrician (YS). The interview data from 15 participants were analyzed using both deductive and inductive approaches to thematic analysis [35, 37]. Deductive analysis was based on Hofstede’ cultural dimensions theory [23]. Specifically, the pre-defined categories developed by Hofstede [23] were used to interpret data related to potential themes regarding the participants’ perceptions of the mentoring relationships from a holistic perspective. Subsequently, the larger themes deductively identified were further examined with a more inductive analysis of the text to generate specific themes that described the participants’ contexts.

This study followed the phases of thematic analysis suggested by Braun and Clarke [35]. The researchers carefully reviewed the transcripts multiple times to better interpret what the participants expressed (i.e., familiarization with data phase). Three researchers (HO, TS, and YS) were independently involved in the initial coding of interview data at both deductive and inductive analytical stages. These researchers then cross-checked their data interpretation and analysis (i.e., coding phase/generating initial themes phase). The preliminary findings were reviewed and discussed by all members of the research team, including RI and KF. In this phase, to refine themes that better reflected participants’ views in given contexts, the existing codes were further revised, and new codes were created if necessary (i.e., reviewing themes phase/defining and naming themes phase). After all researchers came to consensus on all final themes, additional interviews with two more participants were conducted and analyzed to confirm the completeness of the data analysis. All researchers agreed that thematic saturation was achieved at this point.

Credibility of this study was enhanced through triangulation [38, 39]. To achieve data triangulation, this study considered participants with experiences of research fellowship abroad in different countries and data collection was carried out at different times (i.e., after and during research fellowship abroad). Investigator triangulation was also applied. The researchers from different disciplinary and specialty backgrounds worked together to analyze the transcripts throughout the entire data analysis process.

### Ethical considerations

This study was approved by the Institutional Review Board of the Gifu University Graduate School of Medicine (No. 25–96).

## Results

Using Hofstede’s six dimensions of culture, 12 themes in terms of characteristics of mentoring as defined by postgraduate mentees of Japanese physician-scientists were identified in this study (Table 4).

**Table 4:** Characteristic mentoring relationship as defined by postgraduate mentees of Japanese physician-scientists

Cultural dimensions	Themes
Power distance	Trustworthy dependence on mentor Embracing paternalistic mentoring Mentee’s initiative within expectations of mentor
Individualism/collectivism	A sense of loyalty to mentor, organization, or colleagues Family-like relationship with mentor
Uncertainty avoidance	Sense of security on being led by a mentor
Masculinity	Motivation by role modeling for the competitive academic world Adaptation of female mentee/mentor to a male-dominated academic structure
Long-term orientation	A long-term relationship between mentor and mentee Receiving advice for organizational continuity
Indulgence	Putting work before leisure Friendly relationship between mentor and mentee outside of work

### Power distance

#### Trustworthy dependence on mentor

The Japanese physician-scientists had an inner desire to “respect the mentor’s ideas” and naturally accepted the hierarchical relationship and power distance by trusting the mentors. Many of the relationships were mentor-centered, outlining a path for the mentee to follow. As Participant 2 mentioned below, mentees did not have a negative attitude toward the mentor’s suggestions and decisions, but rather, interpreted and accepted them positively and acted upon them due to the natural respect for the experienced senior mentors.

*“When I was asked (by the mentor) what I would like to do at graduate school, I answered ‘I have not decided yet, but I will do anything’, and I left it to him. Then, he told me ‘Leave it to me and wait for a while. I have an idea’. One day, he suggested that I should go to another university laboratory. Throughout my interactions with him, I knew that he was worthy of following, so I was convinced when I received the proposal, and I felt that I got an opportunity.” (Participant 2)*

#### Embracing paternalistic mentoring

Mentees sometimes received harsh guidance and negative feedback from their mentors. However, mentees trusted, appreciated, and embraced the power distance positively. While these words and actions were challenging, mentees perceived that mentors deliberately behaved harshly and negatively to encourage them. For example, Participant 16 said,

*“When I was unable to do the basic things that I should be able to do, he scolded me and said ‘you can do it!’ I believe that this was an intentional method to motivate me. My mentor also advised that I should go and study abroad, in a similar manner. I decided to give it a try.” (Participant 16)*

Receiving both negative and positive feedback on different occasions allowed mentees to accept the harsh behavior as well. For example, Participant 8 said,

*“He (mentor) was a very strict person, and he often reprimanded me. However, he understood what I was doing and praised me when I did well. I thought he was trustworthy and had a good personality.” (Participant 8)*

#### Mentee’s initiative within expectations of mentor

When mentors handed over the initiative or decision making to mentees, mentees still perceived that mentors were superior and that they had a subordinate position. Interview excerpts of Participants 11 and 2 show that mentees felt protected by mentors, expected some consideration and kindness, and tried to meet their expectations.

*“He (mentor) told me that I would be able to study abroad and I could look for the laboratory on my own. I then searched for a disease where my professor's field and my interests overlapped. I found a lab that satisfied both my wishes and my professor’s expectations. In that way, I got my professor's approval and he kindly said, ‘You can go there (as you wish)’. I felt that there were some difficulties in choosing a place unrelated to the professor’s field of interest.” (Participant 11)*

*“To some extent, he gave me flexibility, and I felt that I was protected.” (Participant 2)*

### Individualism versus collectivism

#### A sense of loyalty to mentor, organization, or colleagues

Both mentors and mentees had a strong sense of loyalty toward the research organization or Ikyoku and their colleagues, and they respected the unstated rules of the organization regarding career development. For example, Participant 12 said,

*“I had a friend who was an outstanding researcher and is a professor now. When he studied abroad as a fellow researcher, I thought I would stay at Ikyoku and take care of younger PhD students until he came back.” (Participant 12)*

Most of the individual relationships between mentors and mentees had developed within this framework. The mentees did not seem to be uncomfortable about following the rules of the organization, as loyalty toward the organization was considered a virtue by most of them. For example, Participant 16 said,

*“Basically, I didn't want to go against my mentor or against the policy of our Ikyoku, and it felt natural that I decided my career based on the advice of my mentor. I just followed the advice of the mentor in the Ikoyku.” (Participant 16)*

Individual wishes of mentees, independent from the organizational policies, were accepted by mentors. However, even in such cases, the organizational policies and considerations for colleagues were given importance. For example, Participant 13 said,

*“While I was studying abroad as a postdoctoral fellow, I asked my mentor if I could enter the MBA program alongside the post-doc fellowship and extend my stay in USA. He said ‘It’s okay, but you must complete the MBA even if it takes several years and be sure to get back to Ikyoku’. I appreciated his understanding, and I wanted to get back to Ikyoku when I returned to Japan.” (Participant 13)*

#### Family-like relationship with mentor

Once mentees were a part of the Ikyoku, they received extensive support from mentors, and a strong sense of trust developed. As Participant 8 commented below, the relationship compared with that of a family member, and mentees were entrusting themselves to such private and public relationships with mentors that took place both, inside and outside the workplace.

*“I was invited by the professor and decided to enter the Ikyoku as I had a private conversation with him. He told me, ‘You can consider that I am like your dad’, because he knew that my father passed away when I was a medical student. He was someone I respected, and I was happy to hear that. I feel like he's like my father, like he'll do something for me if the need arises.” (Participant 8)*

### Uncertainty avoidance

#### Sense of security on being led by mentor

Mentees felt reassured as the mentors help them to begin with simple tasks that they were successful at and gradually encouraged them to tackle more complex and difficult tasks. For example, Participant 4 said,

*“He (the mentor) gave me every possible help in learning research methods. I felt bad that I was not very good at research. He was kind enough to help me start at the beginning and also helped me with the laboratory work. He also corrected my dissertation word by word, and it was actually difficult to read it because of the red ink.” (Participant 4)*

While taking important career related decisions, the mentors’ advice, often based on the mission of the organization, was perceived to assist in avoiding uncertainty, and provided a sense of security for mentees, even when it was difficult for them to take the decision on their own. For example, Participant 14 said,

*“When I planned to go to USA, my mentor was worried about me because he was not sure if I was ready to go as I hadn't built up my research foundation yet. He expressed his concern that I would not be able to achieve what I wanted to (and my career would be spoiled or uncertain), and advised me that it would be better to go there after I am an established independent researcher to some extent, in Japan. I felt that his advice was reasonable, and I decided to reconsider my plan.” (Participant 14)*

### Masculinity

#### Motivation by role modeling for the competitive academic world

Mentors, through their words and behaviors, had a strong influence on mentees regarding the importance of work-centeredness and competition. For example, Participant 16 said,

*“When I returned home earlier because I was tired, my mentor worked till quite late and scolded me, saying, ‘You left earlier than me, what's this?’ I was also often scolded in the morning, ‘Why are you coming in after me?’” (Participant 16)*

Although mentors were sometimes strict and rough, mentees took this guidance to heart and became more motivated to succeed in a competitive academic world. Mentees also received guidance from mentors on how to succeed and survive in the academic world. For example, Participant 9 said,

*“(My mentor) was good at setting up rivals. It's like communicating, ‘Don't let that guy beat you’, or ‘He's working hard’. This made me feel that I need to work hard too so that I don't lose to him.” (Participant 9)*

#### Adaptation of female mentee or mentor to a male-dominated academic structure

Female mentees learned from their female mentors how physician scientists of their gender were treated and should behave in a male-dominated academic structure. This made them aware of the unfairness arising from the fact that men were given priority in terms of opportunities, such as promotion and studying abroad. For example, Participant 13 said,

*“I saw that senior female doctors seemed to be very busy trying to balance work and family, and male doctors criticized them for not being able to attend academic conferences or engage in academic activities. Therefore, I realized that female physician-scientists would face such challenges in balancing work and family as well as promotions.” (Participant 13)*

However, they accepted this discrimination and realized that it was something that had to be dealt with; they did not demand any special treatment. Some female mentors also treated female mentees as they treated men and did not give them any special consideration just because they were women. For example, Participant 1 said,

*“My female mentor always put work first, did a great job of nurturing her younger colleagues, and was not the type of person who would give me flexibility just because I am a woman. When it came to choosing a career path, I didn't want to tell her that I was going to follow my husband if he got a position in another hospital.” (Participant 1)*

### Long-term orientation

#### A long-term relationship between mentor and mentee

Mentors tried to create a long-term commitment with mentees and offered advice and guidance regarding their career, including offering tips for academic success (for example, how to expand connection with professors/researchers) and maintaining a good lifestyle (for example, do not be away from the family in a distant place). Mentees also accepted and appreciated the long-term relationship with mentors. For example, Participant 10 said,

*“Our relationship began when I was a student. It's rare to find a medical student who is interested in research, so the professor (mentor) remembered me. He took care of me and told me to come to his department. I wouldn't have been in his department without him.” (Participant 10)*

In particular, when mentees and mentors belonged to the same Ikyoku, mentors would take care of mentees for as long as possible. For example, Participant 4 said,

*“My mentor told me that he was sorry because he had to move to another hospital and he couldn't be my supervisor anymore, even though he invited me to join his department. After a while, when I told him that I wanted to go to graduate school, he looked for a supervisor who would mentor me. Also, he talked to the professor directly and completed all the procedures to make sure I didn't have any trouble later.” (Participant 4)*

#### Receiving advice for organizational continuity

Mentors tried to give advice to mentees for long-term career development and also emphasized long-term future development of organizations and their research projects. Mentees recognized the importance of continuity in terms of organizations and giving benefits to colleagues. They took this advice positively without any sense of discomfort. For example, Participant 14 said,

“*My mentor told me that no one else was participating in the research methodology at our institution, and he said that this was a shame. He asked me if I would go and pursue it (for our laboratory). As I was good at computers, he thought I might be a suitable person for that methodology too.” (Participant 14)*

Mentees felt a natural sense of responsibility as researchers who had been given the opportunity to study abroad. For example, Participant 13 said,

*“As long as I was allowed to study abroad, there was an unspoken understanding that I had to give back to the Ikyoku (and my colleagues).” (Participant 13)*

### Indulgence

#### Putting work before leisure

Mentees continually received guidance from mentors, including the importance of putting work before leisure, even during summer vacations and New Year’s Day. This advice was perceived positively by the mentees. For example, Participant 12 said,

*“I went to the professor’s house many times, not just his office, for guidance regarding my research paper, on weekends. I was called, invited, and asked to come.” (Participant 12)*

Mentees learned that a centeredness toward work and restraint are important mindsets of physician-scientists. For example, Participant 14 said,

*“Working with clinical specimens means that if you miss a chance, you may never get it again. I was imprinted with the mentor’s attitude that I would work if the specimens were available, regardless of whether it was during the summer vacation or New Year's Day, and I have continued to follow this advice.” (Participant 14)*

#### Friendly relationship between mentor and mentee outside of work

Mentors were usually strict and restrained but after work, they would open up to mentees and interact in a relaxed manner. They would go out to eat, drink, and even pursue hobbies together. As Participant 2 commented, mentees also enjoyed the time spent with mentors; they perceived that this interaction was necessary for strengthening the relationship.

*“I was often invited by my mentor to go out for a drink. He enjoyed drinking. I often drank a lot, and my mentor kindly dropped me home. As I often came home late at night from work after drinking with him, my wife would be anxious about what I was doing, but she was okay because she understood our relationship.” (Participant 2)*

## Discussion

This is the first study to qualitatively elucidate the mentoring relationship between senior Japanese physician-scientist mentors and their junior mentees, based on Hofstede’s model of six cultural dimensions as a theoretical framework [23]. It also demonstrates the potential of using Hofstede’s cultural dimensions as a lens through which cultural features of relationships in mentoring can be understood, and suggests the diversity of mentoring in different cultural spheres. Based on a non-Western culture and combined with a referential use of Hofstede’s cultural dimensions and scores, the results could be used as the core of understanding mentoring in various cultures that work collaboratively in the context of globalization. Furthermore, these results could be applied to mentoring in other healthcare fields.

Of these cultural dimensions suggested by Hofstede, power distance, collectivism, uncertainty avoidance, and masculinity were considered particularly relevant to this study. First, mentoring in the presence of a relatively large power distance was positively perceived by Japanese mentees. The power distance score of Japan is 54, which is a medium level; less than that of other East Asian countries such as China and South Korea, but more than that of Western countries [36]. According to Hofstede’s analysis, Japanese people are conscious of their hierarchical position in any social setting and act accordingly [23, 36]. Successful mentoring in Europe and USA is considered to be non-judgmental and the initiative remains with the mentees [40, 41]. The power distance influences their willingness to challenge the instructions [27, 42]. Bossy mentoring was listed as a barrier to mentoring [27]. Thus, from a Western perspective, the paternalistic interaction of mentors seen in this study should be perceived as a hierarchical and unfavorable one that could suppress the mentees’ autonomy [42, 43]. However, the mentees’ perception in this study was not always negative, but such mentoring was perceived as favorable and acceptable in terms of respects for senior experienced mentor. This implies that it would be difficult to understand the mentoring relationship in Japan without taking into consideration the cultural background. Sawatsky et al. investigated mentoring relationships in Malawi, where the power distance score was 70 [44], and identified a similar theme, that is, “respect for elders,” which can be attributed to the cultural values seen in Malawi [19].

Second, individualistic relationships with mentors were rarely mentioned by the mentees in the study. Most of the mentoring relationships were based on a sense of belonging to the organizational group, and a connection with colleagues was frequently mentioned. These are characteristic of collectivism as defined by Hofstede (Table 1) [23]. The individualism score of Japan is 46, which is a medium level, being lower than that of Western countries and higher than other East Asian countries [36]. Individualism has been described as being deeply linked to the success of mentoring as it leads to a focus on the mentee’s career progression as a core goal of the relationship [17, 27], and mentors’ actions are intended to contribute to the mentee’s academic career development and personal growth as an individual [40]. However, in Asian countries, including Japan, that have a collectivism culture, both mentors and mentees consider not only one-to-one relationships but also the influence of the in-group or organization to which they belong. *Bushido: The Soul of Japan*, published in English in 1900, stated that the Japanese placed value on loyalty *(chu-gi)* to the directions and interests of the group (for example, family or company) and placed them above their own needs [45]. Hofstede noted that Japanese persons are famous for their loyalty to their companies and organizations like *Ikyoku,* and discussed that since these can be chosen by people, including mentees, they had an individualistic aspect. However, people in collectivistic countries tend to be loyal to their inner group such as large families and local communities from the beginning [36].

Japan has an uncertainty avoidance score of 92, which is one of the highest values among countries; because of frequent disasters in Japan, people are chary of uncertain situations [36]. Mentees in this study stated that they appreciated the support from mentors, as it could guarantee the certainty of their future career development. In a society that has a strong tendency to avoid uncertainty, people are less likely to change places of employment, and are characterized by security and respect, motivated by affiliation [23]. The reason why seemingly passive career formation was often stated and positively accepted by the mentees in this study was primarily to avoid uncertainty. However, collectivism and long-term orientation should also be considered. Loyalty to a company or an organization, collectivism, lifelong relationships with mentors, and long-term orientation are factors that support career development of mentees and assist in avoiding future uncertainty.

Japan has a score of 95 for masculinity, which is one of the highest values among countries [36]. Through the guidance and support of mentors, mentees of both genders, developed with a strong sense of work-centeredness and awareness regarding academic success. Competition is a universal phenomenon in the academic world. Previous studies from North America have also described competition, though as mainly observed between mentees and mentors in incidents of stealing research achievement or surpassing mentors, which may be based on Western individualism [40, 42]. However, competition between mentees and mentors was rarely mentioned in this study; rather, it was mainly seen between peers or outside the organization. This may be due to trustworthiness and dependency on mentors based on power distance and loyalty to the mentor and organization (Table 4). This study also elaborates on the adaptation of female physician-scientists to the male-dominant academic world. Female mentees would benefit from the advice of mentors of the same gender, as the experience of female physician-scientists would be different from that of male colleagues [46]. Recent studies have identified many challenges faced by women pursuing post-doctoral courses, such as career uncertainly, sexism, maternity leave, and work-life balance [47]. Female mentees in this study were strongly influenced by their female mentors or senior female colleagues regarding their career development. However, female mentors or colleagues seemed to adapt themselves to the male-dominated structure. As female physician-scientists, especially in the senior generation, are a minority in Japan [48], mentoring relationships between male mentors and female mentees are common. Previous research in Japan has identified that female mentees seek mentoring in career development based on the understanding of women’s lives with non-hierarchical relationships, whereas male mentees expect paternalistic mentoring [49].

This indicates that the national culture influences mentoring, and other factors such as organizational culture, generation, and gender should also be considered. Hofstede argues that national culture cannot be altered easily [23] but some studies have claimed that 40 years have passed since Hofstede’s study was conducted and there could have been some changes in this period [50]. In Japan, a post-graduate clinical training system for physicians was introduced in 2006, and since then, a dramatic shift of trainees from university hospitals to general hospitals in the community has been seen [51]. The role of *Ikyoku* and physicians’ perspectives for their career development might change along with this training reform. There is also an increase in the number of female physicians in the younger generation in Japan and women’s value-based mentoring has become more common. This may have an impact on the culture of mentoring.

In a globalized society, the interaction between physician-scientists across the world will increase, and mentoring among different countries, cultures, organizations, and professions will also increase [27]. As culture is an important factor that influences a person’s behavior [52, 53], it is important to understand the cultural impact on mentoring. Although this study was conducted with Japanese physician-scientists to address the relationship between senior physician-scientists and junior physician-scientists, the findings in this study would be useful to ensure culture-sensitive mentoring. Furthermore, this would contribute to nurturing physician-scientists and increase the productivity of research. Additional exploration of how mentoring relationships while abroad differed in contrast to those while in Japan, using more specific questions based on Hofstede’s cultural dimensions, would provide additional important data. Additionally, similar analyses in different cultures could provide a clearer picture of the cultural characteristics of mentoring.

## Limitations

This study has several limitations. First, the number of participants was relatively small and mentors’ views were not addressed. Second, there is a possibility of recall bias among the participants who were reflecting on their mentoring relationships that occurred prior to their studies abroad. Third, we analyzed the data from only one cultural definition or perspective, namely Hofstede’s cultural dimensions, although there are many different definitions of culture. Fourth, we only explored one type of mentoring relationship, namely between senior physician-scientists and junior physician-scientists, and we did not include the various types of mentoring relationships, such as peer mentoring, near-peer mentoring, and group mentoring. Fifth, all the participants (mentees) in this study had graduated from medical school before the new clinical training system was introduced, and this may have influenced their perception. Sixth, the participants (mentees) were mostly males, and this could have influenced the themes based on the cultural dimensions; however, this situation represents the current state of mentoring relationship in Japanese academic medicine.

## Conclusion

This qualitative study revealed the cultural characteristics in mentoring of Japanese physician-scientists and the usefulness of applying Hofstede’s cultural dimensions for understanding mentoring relationships. The findings of this study may assist in ensuring culturally sensitive mentorship henceforth.

## Data Availability

The datasets used and/or analyzed for this study are available with the corresponding author upon reasonable request.
